# Comparative Genomics of Facultative Bacterial Symbionts Isolated from European *Orius* Species Reveals an Ancestral Symbiotic Association

**DOI:** 10.3389/fmicb.2017.01969

**Published:** 2017-10-10

**Authors:** Xiaorui Chen, Matthew D. Hitchings, José E. Mendoza, Virginia Balanza, Paul D. Facey, Paul J. Dyson, Pablo Bielza, Ricardo Del Sol

**Affiliations:** ^1^Institute of Life Science 1, Swansea University Medical School, Swansea University, Swansea, United Kingdom; ^2^Departamento de Producción Vegetal, Escuela Técnica Superior de Ingeniería Agronómica, Universidad Politécnica de Cartagena, Cartagena, Spain

**Keywords:** *Orius*, symbionts, T6SS, *Serratia*, pangenome, biological control

## Abstract

Pest control in agriculture employs diverse strategies, among which the use of predatory insects has steadily increased. The use of several species within the genus *Orius* in pest control is widely spread, particularly in Mediterranean Europe. Commercial mass rearing of predatory insects is costly, and research efforts have concentrated on diet manipulation and selective breeding to reduce costs and improve efficacy. The characterisation and contribution of microbial symbionts to *Orius* sp. fitness, behaviour, and potential impact on human health has been neglected. This paper provides the first genome sequence level description of the predominant culturable facultative bacterial symbionts associated with five *Orius* species (*O. laevigatus, O. niger, O. pallidicornis, O. majusculus*, and *O. albidipennis*) from several geographical locations. Two types of symbionts were broadly classified as members of the genera *Serratia* and *Leucobacter*, while a third constitutes a new genus within the Erwiniaceae. These symbionts were found to colonise all the insect specimens tested, which evidenced an ancestral symbiotic association between these bacteria and the genus *Orius*. Pangenome analyses of the *Serratia* sp. isolates offered clues linking Type VI secretion system effector–immunity proteins from the Tai4 sub-family to the symbiotic lifestyle.

## Introduction

The food needs of an ever-increasing human population must be matched by intensification of crop production, albeit using sustainable approaches to ensure the preservation of ecosystems, biodiversity, and environmental quality. Pest control is a key element in food production and with thousands of described crops pests (mainly weeds, invertebrates, and plant pathogens), a multitude of pest management strategies have been developed throughout human history to minimise damage and reduce crop losses. Although chemical pesticides were considered at one point a “silver bullet” for pest control, unwanted side effects for both human and environmental health have prompted the search for alternative approaches (Chandler et al., 2011).

Integrated pest management (IPM) strategies have evolved to address the need for a systems approach combining crop protection strategies with the monitoring of agricultural pests and their natural enemies. The use of insect predators as biological control agents has ancient origins, and their use has gathered popularity within the context of IPM programmes as a more environmentally safe and economically viable pest management method. Several types of biological control are in use (natural, conservation, inoculative, and augmentative) – with the latter consisting of mass rearing of indigenous or exotic predators on a semi-industrial scale. Indeed, augmentative biological control is now a successful commercial activity (>$200 million) with more than 200 predatory species (mostly Arthropoda) produced and sold by numerous suppliers to be used in field and greenhouse grown crops. Among the predators used, the family Anthocoridae (Hemiptera: Heteroptera) is well represented by several species within the genus *Orius* (Wolff), most of them with a substantial market presence since the 1990s. Of those, the generalist predator *Orius laevigatus* in particular is currently used in over 20 countries as a pest control agent (van Lenteren, 2012).

There are more than 70 species of the genus *Orius*, most commonly distributed in the Palearctic, but also described in the Nearctic and Neotropic realms (Horton et al., 2016). Most species are predaceous, although many display facultative phytophagy to varying degrees with *Orius pallidicornis* (Reuter) currently the only known exception; feeding primarily on pollen of *Ecballium elaterium* (Lattin, 1999; Péricart, 1972). Several *Orius* species are currently used to control global pests (e.g., *Frankliniella occidentalis, Thrips palmi*, and *Thrips tabaci*). *O. sauteri* (Poppius) and *O. strigicollis* (Poppius), among others, have been used in Japan as biological control agents (Yano, 2004). In Europe, the use of *Orius* sp. is widespread, particularly in Mediterranean countries where native populations of *O. majusculus* (Reuter), *O. laevigatus* (Fieber), *Orius albidipennis* (Reuter), *Orius niger* (Wolff), *Orius minutus* (L.), *Orius horvathi* (Reuter), and *Orius laticollis* (Reuter) do occur (Gomez-Polo et al., 2013). Thus, the presence of indigenous *Orius* species and their effectiveness in controlling insect pests have driven efforts to understand their ecology within the predator–prey interaction context, and to reduce the cost of artificial rearing to expand their use in IPM applications. However, most of the published work relates to the molecular taxonomy of *Orius* sp. to monitor field persistence and efficacy, or is focused on artificial diet manipulation. Indeed, animal prey seem to be an essential dietary component for optimal development for *O. albidipennis* and *O. laevigatus* (Vacante et al., 1997) – this is particularly relevant for the latter as its production costs are high due to the use of *Ephestia kuehniella* eggs as food source for mass rearing (Bonte and De Clercq, 2008).

Despite their extensive use in IPM, there is a conspicuous absence of studies exploring the role of microbial symbionts on development, speciation, fitness, and behaviour of *Orius* species. Broadly, symbiosis is classified as mutualism, commensalism, or parasitism, and this definition is used throughout this paper. Symbiotic relationships between insects and microorganisms are ubiquitous, and based on the establishment of persistent infections in the host that are vertically (maternally) or horizontally transmitted. The nature of the symbiont–host relationship defines two categories of symbiosis: primary or obligate symbionts and facultative or secondary ones. Primary symbionts are essential for host survival and share a long evolutionary history with the host, as evidenced by the 270 million years association between *Sulcia muelleri* and *Auchenorrhyncha* (Ferrari and Vavre, 2011). They are usually confined to specialised organs (bacteriome) and confer nutritional benefits to the host in the form of essential amino acid or vitamin biosynthesis. The best characterised example is the association between aphids and their obligate symbiont *Buchnera aphidicola* whereby essential amino acids absent from the host’s diet are provided by a symbiont that, as a result of genome shrinkage, depends entirely on the host for many metabolites (Dale and Moran, 2006; Ferrari and Vavre, 2011).

Facultative symbionts on the other hand establish mutualistic relationships and generally are not essential for host’s survival or development, although many contribute to overall fitness (Whitten and Dyson, 2017). They may infect various tissues, with maternal and/or horizontal transmission common; even across diverse arthropod lineages (Russell et al., 2003). Typical examples of this group are reproductive manipulators (*Wolbachia, Spiroplasma*, and *Cardinium* species), symbionts that confer protection to parasitoids (*Hamiltonella defensa* in aphids), or fungal infection (*Regiella insecticola, Streptomyces* sp.), and to environmental stress (*Serratia symbiotica* in aphids). The latter putatively provides evidence of the early stages in the transition from facultative to primary symbiosis, as it likely complements essential functions lost by the primary symbiont *Buchnera* in the aphid *Cinara cedri* (Moran et al., 2008; Ferrari and Vavre, 2011; Manzano-Marín and Latorre, 2016). Among the insect tissues or organs colonised by symbionts, the gut has been extensively studied as it hosts a diverse microbiome, composed of essential and facultative symbionts that coexist and compete with transient commensals and pathogens acquired from the environment (Engel and Moran, 2013). Expanding our understanding of insects’ facultative symbionts not only provides a better view of their role in host ecology, but provides access to untapped biotechnology resources supporting pest control strategies or the discovery of novel biomedical compounds (Berasategui et al., 2016; Whitten et al., 2016).

The study of microorganisms associated to *Orius* species is limited, with only two studies (utilising culture-independent approaches) being published. Of those studies, *Wolbachia* infection was detected using multilocus sequence typing (MLST) profiling in multiple populations of five *Orius* species (*O. sauteri, O. nagaii, O. minutus, O. strigicollis*, and *O. tantillus*) from Japan. Only two predominant *Wolbachia* strain types were detected in the study, which prompted the conclusion that both vertical transmission from a common ancestor and horizontal transfer account for the low diversity of *Wolbachia* lineages observed (Watanabe et al., 2011, 2012, 2014). A study using the above insect specimen cohort described the co-infection of *Wolbachia* and *Spiroplasma*. Although the study concluded that the latter is transmitted vertically, the presence of *Spiroplasma* in a biocontrol agent such as *Orius* is concerning, due to the numerous *Spiroplasma* species recognised as plant pathogens (Watanabe et al., 2014).

There is a clear knowledge gap in our understanding of culturable microorganisms associated with *Orius* species. This is especially relevant for IPM practises, and the need to implement quality control procedures that take into account the microbiome’s influence on physiology and evolution of a mass-produced pest control agent. The boundaries between pathogenic and mutualistic microorganisms are often unclear, in particular for facultative symbionts that can be horizontally transferred between prey and predators, and from these to the plants the insects inhabit and humans consume. This paper provides the first comparative genomics report on the predominant culturable symbionts associated with several populations of *O. laevigatus* and other European *Orius* species, offering a glimpse at facultative microbial populations associated with this important pest control agent. Our observations show that the association of European *Orius* sp. and their predominant facultative symbionts is not host species specific, indicating a probable ancestral association that likely predates hosts speciation. Furthermore, pangenome analyses in one of the *Orius* symbionts suggest that Type VI secretion genes encoding for effector–immunity cognate proteins reflect the needs arising from a symbiotic lifestyle.

## Materials and Methods

### Insect Specimens

*Orius laevigatus* populations, as well as other *Orius* species assayed (Supplementary Table 1), were collected using a hand aspirator from multiple regions of the Mediterranean Basin (September 2012 and May 2016), on different indigenous plant species far from commercial release areas. A proportion of the specimens were preserved in 70% ethanol while the rest was reared for two generations in the laboratory ensuring segregation according to source population geographical location.

Rearing and husbandry of collected insects was performed in Cartagena (Murcia, Spain) at the Crop Protection laboratory (Universidad Politécnica de Cartagena). These procedures were carried out in previously sterilised, 1 L plastic containers with tight fitting lids containing air vents covered in philtre paper. Sterilised buckwheat husk (*Fagopyrum*) was used as a refuge; frozen and sterilised *E. kuehniella* eggs were used as a prey for nutrition; surfaced sterilised pods of *Phaseolus vulgaris* (with the ends sealed using paraffin wax) were provided for egg laying; and a water vial sealed with cotton wool as a source of moisture. Containers were maintained at 25–26°C, in 70–80% relative humidity and 16L:8D daylight cycle. Maintenance and multiplication of these populations were undertaken every 2 or 3 days to collect *Orius* eggs, launching a new age cohort of insects and avoiding cannibalism from adults to nymphs. Moreover, *Ephestia* eggs and a fresh bean pod piece were added each time, water vials were replaced as necessary. Four commercial *O. laevigatus* specimens were purchased from Syngenta Bioline^®^, Koppert (THRIPOR-L), BioBest (Orius-System), and AgroBio (Oricontrol). *O. pallidicornis* specimens were collected in the field (Southeast Spain) and processed immediately or preserved in 70% ethanol. Taxonomic classification of insect specimens was confirmed by amplifying COI by PCR from total insect DNA using primers CoxILCO1490F and CoxIHCO2198R (Folmer et al., 1994), followed by amplicon sequencing and homology comparison to databases.

### Isolation of Culturable Bacteria from Insect Specimens

Live insects (∼10 individuals) were surface sterilised twice with 70% ethanol, washed in sterile water three times, and crushed using sterile micropestles in liquid Nutrient Broth. The homogenate was diluted in Nutrient broth and plated on Nutrient Agar plates using aseptic techniques, followed by incubation at 28°C until colonies were visible (2–3 days). *Ephestia* eggs, bean pods, and buckwheat husk were processed similarly to determine their bacterial content. Bacterial colonies that emerged after 2–3 days were roughly classified based on morphology, and predominant morphotypes were streaked on Nutrient Agar to ensure culture purity, followed by liquid culture to prepare 40% glycerol stocks for preservation. Colony PCR (of 16S rRNA gene) was performed on the selected colony morphotypes and the resultant amplicon sequenced to confirm taxonomic identity Primers used here were U1 and U1R (James et al., 2010).

### DNA Extraction and Genome Sequencing

Total insect DNA was isolated from surfaced sterilised insect specimens, using the DNeasy^®^ Blood & Tissue Kit (insect protocol) from Qiagen. To increase yield, specimens were incubated in Proteinase K overnight. Total bacterial DNA was isolated from overnight Nutrient Broth cultures using a similar kit applying the corresponding protocol (Qiagen). Total genomic DNA was quality controlled by electrophoresis and spectrophotometry. Bacterial genome sequencing was performed on an Illumina MiSeq platform. Sequence ready libraries were constructed using the Illumina Nextera XT library preparation procedure. Raw reads were subjected to quality and adapter filtering using the Trim Galore wrapper tool specifying the Illumina and Nextera adapter strings to be trimmed (Martin, 2011). Contiguous sequences were *de novo* assembled using the SPAdes v3.5.0 assembler (Bankevich et al., 2012) and contigs below 500 bp were filtered out before the final assembly evaluation was carried out using QUAST (Gurevich et al., 2013). Draught genome sequences were annotated using the Prokaryote Genome Annotation Pipeline (PGAP; Tatusova et al., 2016).

### Detection of Symbionts Using Genome-Specific PCR

Annotated ORFs from representative genomes were used to query all available NCBI DNA databases using BLASTN (Altschul et al., 1990). Those ORFs not possessing a homologous sequence in any other bacterial genome, including those reported in this paper, were further filtered to remove ORFs encoding for mobile elements (plasmids, phage, and transposable units) or nucleotide length below 300 bp. The selected genome-specific ORFs were used as template to design PCR primers with Primer BLAST (Ye et al., 2012). PCR primers used in this study are listed in Supplementary Table 2. PCRs were performed using 1 μL of total insect DNA in 20 μL reaction volumes (Supplementary Methods). Amplicon identity was confirmed by sequencing. DNA extracted from *E. kuehniella* eggs was used as negative control.

### COI Phylogeny Methods

Phylogenetic reconstruction of *Orius* species was performed using a maximum-likelihood (ML) phylogeny. Briefly, COI sequences were amplified using primers CoxILCO1490F and CoxIHCO2198R. Resultant amplicons were precipitated overnight and sequenced in both directions using the original PCR primers. Consensus COI sequences were constructed by alignment of both forward and reverse sequences. Orthologous COI sequences from other *Orius* species were retrieved from GenBank and also added to the dataset. ML phylogenetic reconstruction was performed in MEGA using the Tamura-Nei model and uniform rates among sites. Robustness of the phylogeny was assessed with 1000 bootstrap pseudoreplicates.

### Genome Sequence Analyses

Phylogenetic placement of bacterial symbionts isolated from *Orius* sp. was performed using a multilocus amino acid phylogeny. Briefly, ortholog retrieval, alignment, and concatenation of 400 ubiquitous and phylogenetically informative protein sequences were performed in PhyloPhlAn (Segata et al., 2013). In addition to our isolates, we included translated CDS files (.faa) from all Enterobacteriale and Actinobacterial genomes retrieved autonomously from the GenBank FTP site (last accessed September 2016). Concatenated alignments were used to generate unrooted ML phylogenies in FastTree MP (Price et al., 2010) implemented in the Cipres Science Gateway Server (Miller et al., 2010). Trees were reconstructed using the JTT+CAT model with 20 discrete categories (-cat 20). Topology refinement was performed using the following parameters: -nni 10 -spr 2 -sprlength 10. Nodal support was inferred from 1000 bootstrap pseudoreplicates.

Species delimitation was further determined by performing pairwise digital DNA:DNA hybridisation to calculate genome-to-genome distances (GGDC) using GGDC 2^1^, and a distance threshold of 70% as recommended with Formula 2 for draught genomes (Meier-Kolthoff et al., 2013).

### GI Prediction

Horizontally transferred genomic islands were predicted using IslandViewer 3 (Dhillon et al., 2015) using *Serratia* sp. SCBI as a reference. Predicted Genomic Islands (GIs) sequences were clustered using CD-Hit-est^2^ using an 80% identity threshold in order to identify representative GIs (Supplementary Table 7). A hierarchical clustering analysis was carried out using the presence of clustered GIs to assess the relatedness of the isolates based on genomic GI content. The dendrogram and binary matrix were visualised using iTOL (Letunic and Bork, 2016).

### Pangenome Analysis

Bacterial genomes identified within this study as belonging to the same species were annotated using PROKKA (Seemann, 2014) and processed in a pangenome pipeline ROARY (Page et al., 2015). Briefly, minimum sequence length was set to 120 bp and only sequences containing both start and stop codons were used. Iterative clustering steps starting at 100% identity and 100% length and reducing in 0.5% steps down to 98% were used to remove core genes present in all isolates. A gene is considered part of the core genome if it occurs at least once in every isolate with a 95% sequence identity. Evoking the -r option returns the results of random and iterative addition of isolate genomes to the pangenome analysis in order to identify if the pangenome is of an open or closed nature. A pangenome wide association study (pan-GWAS) was carried out using SCOARY (Brynildsrud et al., 2016) to associate gene presence or absence with the trait of facultative insect symbiosis. Genes were considered associated to insect symbiosis if they were present only in all insect facultative symbionts. Additional annotation was carried out using HMMER (Finn et al., 2011).

## Results

### Isolation of Facultative Symbionts from *Orius* Populations

**FIGURE 1:**
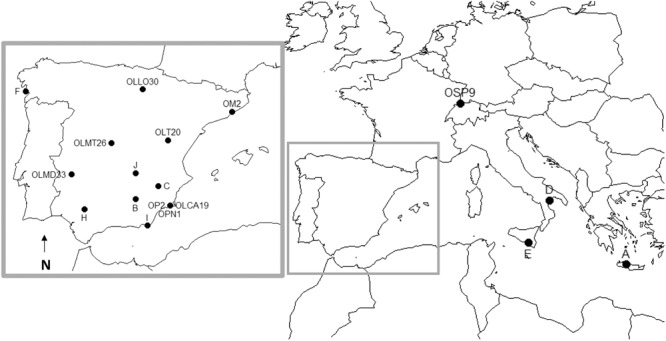
Geographical locations where insect specimens were collected to establish lab-reared populations. *O. pallidicornis* collection sites are also shown. Description of populations is shown in Supplementary Table 1. *O. laevigatus* (A, B, C, D, E, F, H, I, J, OLLO30, OLMT26, OLT20, OLMD33, and OLCA19), *O. majusculus* (OM2), *O. niger* (OSP9), and *O. pallidicornis* (OPN1, OP2).

Field collected insect specimens were taxonomically classified using morphological characteristics (Ferragut and González-Zamora, 1994; Supplementary Table 1) and preserved in 70% ethanol or propagated in the lab to establish populations, segregated according to their original geographical location, as indicated in **Figure 1**. Of particular note is the inclusion in this study of *O. pallidicornis* specimens. This species cannot be reared in the lab due to its requirement to feed on pollen from *E. elaterium*; therefore, only field collected specimens were used. The initial taxonomic classification of insects was confirmed by COI sequence phylogeny, using representative COI sequences from a variety of *Orius* species (Supplementary Figure 1). The COI-derived phylogenies grouped all specimens used within this study according to their predicted genealogy. Interestingly, our analysis grouped *O. pallidicornis* and *O. albidipennis* as independent monophyletic lineages. This is important as this is the first study to provide any sequence data for these two *Orius* species – and thus confirms their status as unique species. Additionally, in our analysis *O. niger* is represented by two well-defined clades with the *O. pallidicornis* clade as a sister grouping, next to one of the two *O. niger* clades. This dichotomy is indicative of at least two sub-species of *O. niger*. The placement of the *O. pallidicornis* clade suggests a close evolutionary relationship between these two species – hinting at *O. pallidicornis* and *O. niger* being derived from a recent speciation event. The *O. minutus* distribution is irregular; suggesting that a revision of the taxonomic classification of the specimens used to obtain the COI sequences is required.

Plating of whole insect homogenates, prepared from surface sterilised specimens, resulted in the growth of different combinations of three predominant bacterial colony morphologies across the range of samples tested. The three colony morphologies were not cultured in similar abundance levels from any homogenate. Representative colony types from each insect population sampled were used as template for 16S rRNA gene amplification, leading to the classification of 10 isolates belonging to Actinobacteria (*Leucobacter*-like). The rest of the isolates (24) belonged to *Enterobacteriales* (15 *Serratia-*like, 8 *Erwinia-*like, and one related to *Tatumella*; Supplementary Table 3).

### Phylogenomic Analyses of *Orius* sp. Facultative Symbionts Identified Three Putative New Species

The whole-genome sequences of 34 isolates were obtained, assembled, and annotated as described (Supplementary Table 4). Due to the limitations of 16S rRNA gene-based phylogeny; a multilocus sequence alignment (MLSA) approach was used to accurately classify the isolated symbionts. Orthologous protein sequences (400) from all publically available *Enterobacteriales* and *Actinobacteria* genome sequences were retrieved and used to generate a concatenated sequence alignment using PhyloPhlAn. This multilocus concatenated alignment was used to generate a ML phylogenetic tree, leading to a more refined taxonomic classification of the different facultative symbiotic species associated to *Orius* species.

**FIGURE 2:**
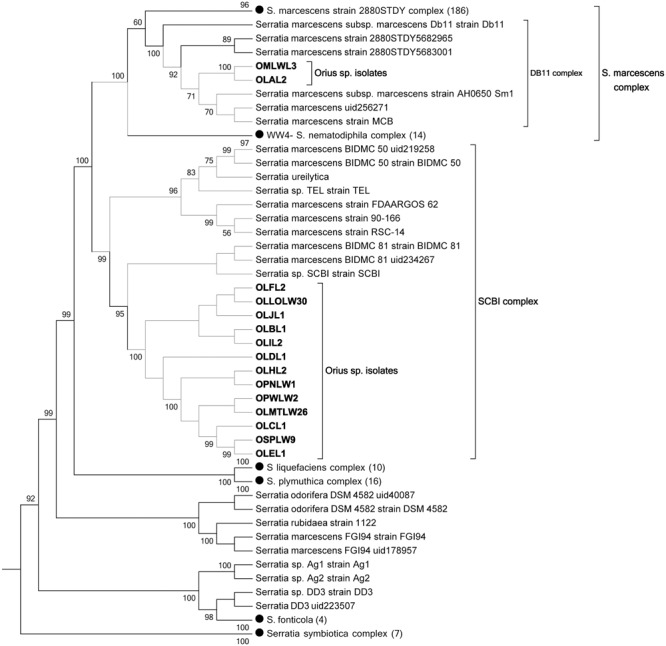
Multilocus phylogeny of *Serratia*-like facultative symbionts isolated from *Orius* species. Topology bootstrap consensus tree (PhyloPhlan) is shown, using collapsed nodes (black dots) for simplicity, with number of genomes per node indicated within brackets.

The 15 *Serratia*-like isolates distributed into two strongly supported clades, predominantly populated by *Serratia marcescens*. Several species within these clades are not classified as *S. marcescens*, suggesting that a phylogenomics-based revision of the taxonomic classification of this genus is due. We refer to these clades as “*S. marcescens* clade” and “*Serratia* SCBI clade” to simplify the discussion (**Figure 2**). Thirteen *Orius*-derived isolates distributed to a tight small clade together with 10 *Serratia* species within the “*Serratia* SCBI complex,” while two isolates grouped with the reference strain *S. marcescens* DB11 in a smaller clade (DB11 complex) within the major “*S. marcescens* complex.” Further genome–genome distance calculation (GGDC) analysis of the genome sequences present in the “*Serratia* SCBI clade,” using digital DNA:DNA hybridisation confirmed that all genomes in this clade belong to the same species and will be referred to as *Serratia* SCBI-like isolates (data not shown). However, this analysis also showed that this sequence similarity is not sufficient to consider the *Orius*-derived isolates the same sub-species as those within the SCBI clade (Supplementary Table 5). Interestingly, when compared to genomes from the “*S. marcescens* complex” including the *Orius*-derived genomes OLAL2 and OMLWL3, the genome sequence distance fell below the same species threshold, indicating that the species conforming the SCBI clade are indeed a different *Serratia* species closely related to *S. marcescens*.

Since some of the organisms grouping within the SCBI complex are classified as *S. marcescens* sub-species (**Figure 2**), the accuracy of the *S. marcescens* taxonomic classification in some genomes seems questionable. Therefore, genome distance comparisons using as query *S. marcescens* WWW4 to all available fully assembled genomes of bacteria classified as *S. marcescens* in the NCBI nucleotide database were conducted, revealing in some cases (e.g., *S. marcescens* DB11, *S. marcescens* SM39, *S. marcescens* FGI94) genome sequence distances that disprove their taxonomic classification as the same species (data not shown).

**FIGURE 3:**
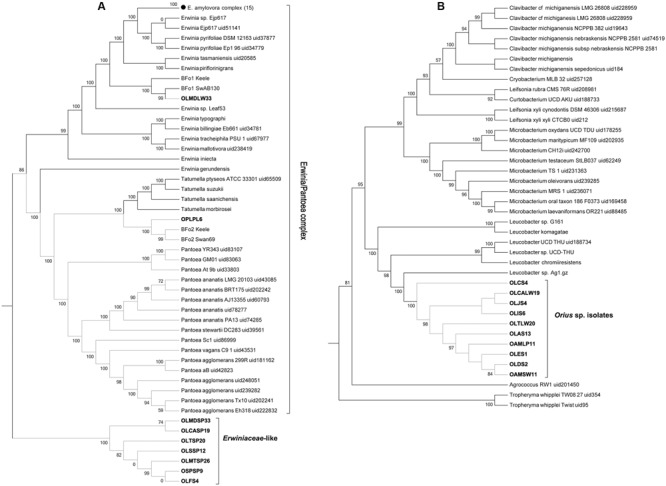
Multilocus phylogenetic distribution of *Orius*-derived *Erwiniaceae*-like **(A)** and *Leucobacter*-like **(B)** isolates, obtained using PhyloPhlan. Collapsed nodes were used for simplicity, with number of genomes per node indicated within brackets.

The genome sequences from the *Erwinia*-like and *Leucobacter*-like isolates were processed in a similar manner using PhyloPhlan. From the former, only one isolate (OLMDLW33) grouped within the *Erwinia*/*Pantoea sensu stricta*, forming a small clade with the *F. occidentalis* symbiont BFo1 (Facey et al., 2015). This distribution was confirmed by GGDC comparisons, which showed that OLMDLW33 and BFo1 belong to the same species. The isolate OPLPL6 from *O. pallidicornis* grouped with the other main *F. occidentalis* symbiont BFo2, although it displayed 68% probability by GGDC comparison of being the same species as BFo2 (i.e., similarity <70% threshold). Surprisingly, the remaining seven “*Erwinia*-like” isolates disputed the 16S rRNA gene-based classification and distributed to a novel monophyletic clade within the Erwiniaceae (**Figure 3A**). The value of this distribution was confirmed by GGDC analyses, as it showed that our isolates were too distant from any of the available *Erwinia, Pantoea*, or *Tatumella* species genomes; probably constituting a new genus yet to be properly classified. Further attempts with GGDC analyses failed to identify a close relative in publicly available genomes, and from this point these isolates are referred to as Erwiniaceae*-*like.

The *Leucobacter*-like isolates formed a strongly supported novel monophyletic clade within the Microbacteriaceae, as part of the *Leucobacter* clade (**Figure 3B**). Despite this distribution and the 16S rRNA gene sequence similarity, genome distance comparisons estimated by GGDC revealed that none of the isolates were similar enough to be considered the same species as the *Leucobacter* sp. genomes available at NCBI databases. These genomes are referred to as *Leucobacter* sp. Orius throughout the paper. Further exploration identified one additional *Leucobacter* sp. genome sequence, namely *Leucobacter* sp. AEAR, which was assembled from the raw genome sequences of the nematodes *Caenorhabditis angaria* and *Caenorhabditis remanei*, although never isolated and cultured (Percudani, 2013). When compared by GGDC, *Leucobacter* sp. AEAR genome was shown to be similar enough to be considered the same species to the *Leucobacter* sp. Orius isolates, and together they should be considered a new species within the *Leucobacter* genus. Despite this similarity, comparison of genome statistics revealed differences, mostly in terms of number of coding sequences (*Leucobacter* sp. AEAR: 2778, Leucobacter sp. Orius: ∼3010 ± 189) that suggest different sub-species. Indeed, while GGDC produced a high probability score (82.34%, higher than the 70% threshold) confirming that these genomes belong to the same species, the probability of them being the same sub-species (33.31%) falls well below the defined threshold (>79%).

### Genome-Specific PCR Confirms the Association of *Orius* sp. Hosts and Their Facultative Symbionts

Representative genome sequences from the distinct isolates described above were used to design genome-specific PCR primers (Supplementary Table 2). These primers were used to detect the presence of the corresponding target sequences in total DNA extracted from insect specimens used as source of isolates. Since all but one (*O. pallidicornis*) insect species tested were lab-reared, total insect DNA was also extracted from specimens collected in the field or acquired from commercial sources, but not propagated in the lab. Genome-specific PCR amplification detected the presence of *Orius*-derived *Serratia* SCBI-like strains in insect specimens from where *Serratia*-like isolates were not recovered by culturing techniques; and also from field collected or non-lab reared specimens (Supplementary Figure 2A). *F. occidentalis* total DNA was used as template in a similar control experiment and no PCR product was amplified (not shown). The *Serratia* isolates belonging to the DB11 complex (OLAL2 and OMLW3) were not detected in any of the insect DNA samples tested (not shown), and therefore it was concluded that there was insufficient evidence to catalogue these isolates as true symbionts, and were not further analysed.

Similarly, the presence of the Erwiniaceae-like organism was confirmed across several *Orius* species in most of the specimens tested, including none lab-reared hosts like *O. pallidicornis* (OPN1, OP18516). In contrast, the Erwiniaceae-like organism was only detected in lab-reared *O. albidipennis* and not detected in some commercial specimens (Supplementary Figure 2B).

Attempts to detect the presence of the *Leucobacter* sp. Orius strains using a similar approach were successful in lab-reared specimens, but not from field collected or commercially purchased ones (results not shown), questioning the symbiotic association of these isolates with *Orius* sp. This finding, together with the limited number of genome sequences from the same species, led to the decision of not performing further analyses on these strains until their symbiotic nature is confirmed by future studies.

### Genomic Island Predictions Differentiate *Orius*-Associated *Serratia* Strains

Genomic Islands are gene clusters arising from horizontal gene transfer events, usually resulting in the acquisition of genetic traits advantageous for the colonisation diverse ecological niches. As such, GIs are powerful drivers of evolution. Additionally, GI prediction is a suitable tool to assess genome plasticity and to segregate closely related strains. The GI content of the *Serratia* SCBI-like genomes was predicted in Island Viewer using *Serratia* SCBI as reference genome. Overall 87 GIs were predicted, with sizes ranging from 50 to 262 Kb (Supplementary Table 6). Interestingly, the number and length of GIs per genome vary, ranging from 3 GIs in OLJL1 to 11 in OSPLW9. Most of the *O. laevigatus*-derived isolates displayed a comparable number of GIs (ranging from 5 to 9), with the exception of OLFL2 (4) and OLJL1 (3). Overall there is poor correlation between genome size and GI number (Supplementary Figure 3A). Unexpectedly, a small correlation was observed between GI number and length (Supplementary Figure 3B), triggered by outliers like OPWLW2 (7 GIs, 262 Kb) and OSPLW9 (11 GIs, 145 Kb). As expected most of the GIs identified encode for mobile elements (Supplementary Table 7).

**FIGURE 4:**
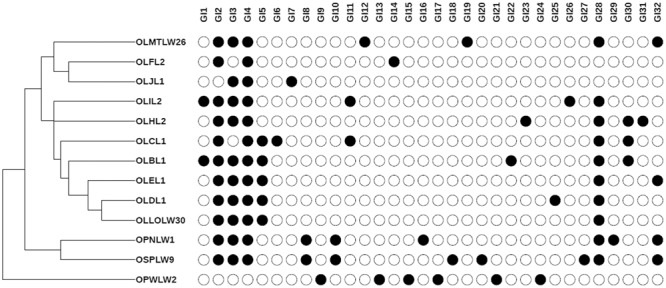
Genomic Islands profiles in *Orius* derived, *Serratia-*like facultative symbionts. Presence–absence map showing the distribution of representative GIs per genome (C). GI number corresponds to those described in Supplementary Table 7. Dendogram generated based on GI presence/absence per genome.

The GI sequences were clustered into representative GIs (32 unique GIs, ≥80% identity, ≥30% query coverage) to identify those shared across genomes from genome-specific GIs (Supplementary Table 7). While some are shared across most genomes, a large number of predicted GIs were genome specific, indicating that the isolates constitute independent strains. As expected, *O. laevigatus-*derived isolates shared numerous GIs, while *O. niger*-(OSP9) and *O. pallidicornis* (OPN1, OPWLW2)-derived isolates possess, respectively, a large number of genome-specific GIs (**Figure 4**). Interestingly, the distribution of these insect hosts based on presence/absence of representative GIs in genomes of corresponding symbionts is reminiscent of the COI-based phylogenetic relationship of *O. niger* and *O. pallidicornis*, as proposed earlier. The lack of available reference genomes for the Erwiniaceae-like and *Leucobacter* sp. Orius prevented the prediction of GIs in the genomes of these isolates.

### Pangenome Analysis of the *Serratia* SCBI-Like Genomes Reveals Insect-Associated Genes

The absence in databases of fully assembled reference genomes from the same species as the *Leucobacter* sp. Orius and Erwiniaceae-like isolates determined that pangenome analyses were limited to *Serratia* SCBI-like genomes. All genomes in the “*Serratia* SCBI complex” were included in the dataset, and processed using ROARY. Except *Serratia* sp. SCBI, all genomes in this complex are draught genomes not segregated into replicons, and are therefore likely to contain plasmid sequence(s) as part of the assemblies. In order to ensure a meaningful comparison, before undertaking pangenome analysis the sequence of the *Serratia* sp. SCBI plasmid (SCBI_Pl) was included as part of this organism’s genome sequence.

Comparative analysis of the gene content between the 21 genomes within the “*Serratia* SCBI complex” reveals that the pangenome contains a total of 8165 genes with some 43.1% of genes shared across all isolates – yielding a core genome of 3518 genes. The accessory genome can be sub-divided into soft core genes (95% ≤ strains < 99%), shell genes (15% ≤ strains < 95%), or cloud genes (0% ≤ strains < 15%) of which there are 515, 1160, and 2972 genes, respectively.

**FIGURE 5:**
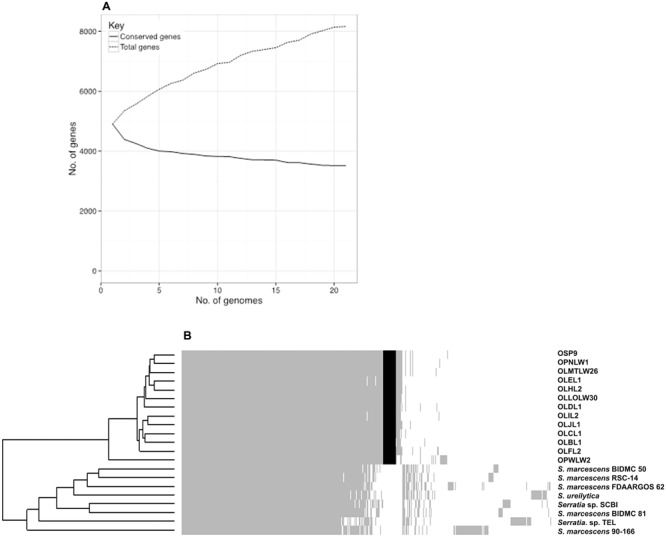
Pangenome analysis of strains within the *Serratia* SCBI complex. Overview of the complete pangenome displays stabilising conserved gene content while total gene content continues to augment, suggestive of an open pangenome for the SCBI complex **(A)**. The pangenome gene presence/absence metrics displayed as a heatmap highlights the diversity within the SCBI complex. Grey shading confirms gene presence, white space confirms gene absence, and black shading highlights all *Orius*-associated genes absent from all other members of the SCBI complex **(B)**.

Sequencing of additional members of this *Serratia* SCBI complex is unlikely to significantly alter the estimated core genome size; on the other hand, the complete pangenome is likely to increase in size as additional members of the SCBI complex are sequenced (**Figure 5A**), indicating an open pangenome for species within this complex. A total of 279 accessory genes were found to be strictly associated with the *Orius* facultative symbiosis trait, occurring in all 13 *Orius*-derived isolates but not in any of the other strains from the SCBI complex (**Figure 5B**).

Annotation of the predicted *Orius* symbionts’ accessory genome (referred as *Orius* accessory genes) revealed a high number of hypothetical proteins, transcription factors, bacteriophage proteins, mobile elements, and seemingly plasmid-related genes (Supplementary Table 8). The latter is at odds with the inclusion of the *Serratia* sp. SCBI plasmid within the analysis. Sequence homology searches using these plasmid-related genes identified single contig assemblies corresponding to putative plasmid sequences, well conserved across the *Serratia* sp. draught genomes reported here. A representative sequence (NODE_31 in OLFL2, GeneBank SAMN05868420) was used as query in BLASTN to search available *Serratia* sequences, disclosing that these plasmid sequences possess higher sequence homology to plasmids PSM22 (*S. marcescens strain* B-6493) and PWN146p1 (*S. marcescens* PWN146) than to SCBI_P1. This explains why some plasmid genes are included within the *Orius* accessory genes. *S. marcescens strain* B-6493 is a human pathogen and *S. marcescens* PWN146 was isolated from the nematode *Bursaphelenchus xylophilus*, and both are different species from those in the SCBI complex as confirmed by GGDC. With a lack of replicon segregation within available *Serratia* species draught genome assemblies, it is difficult to expand on plasmid sequence comparisons at this stage to define a plasmid genealogy for the genus, but our findings are certainly indicative of plasmid exchange and sequence plasticity across the *Serratia* genus and worthy of future studies.

**FIGURE 6:**
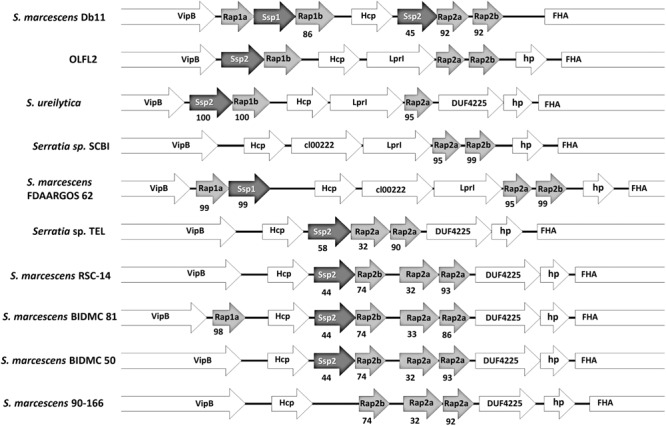
Synteny map of the Ssp–Rap locus in *Orius*-associated *Serratia* (OLFL2), compared to strains from the *Serratia* SCBI complex and *S. marcescens* DB11. Protein product names are shown, and sequence identity to corresponding ortholog in OLFL2 or *S. marcescens* DB11 is indicated below each gene. Well-conserved genes (VipB, Hcp, and Fha) are also shown. Uncharacterised encoded proteins are shown as hypothetical protein (hp) or named based on predicted conserved domains content (LprI: cl26926, DUF4225, cl00222).

Interestingly, within the *Orius* accessory genome a Type VI secretion system (TSS6) associated protein was identified, and annotated as an immunity protein belonging to the Tai4 protein family. Considering the implication of TSS6 in bacterial virulence and inter-bacterial competition, the TSS6-related genes in the genomes analysed were scrutinised. The *Orius*-derived *Serratia* genomes possess two TSS6 genetic loci, with most of the genes in these loci contained within the SCBI core genome, with the exception of the aforementioned Tai4-like encoding gene identified as part of the *Orius* accessory genome. The TSS6 locus encoding this protein was further analysed; revealing the presence of a well-conserved TSS6 gene cluster. It is worth noting that the PGAP annotation pipeline failed to predict some of the ORFs in this locus that were otherwise identified by the PROKKA annotation pipeline, thus these annotations were used to map the genes. In addition to the conserved core TSS6 genes, several genes encoding for effector and immunity cognate partners from the Tae4/Tai4 subfamily were identified. Tae4-like proteins are bacterial cell wall targeting amidases, whose activity is neutralised by cognate Tai4-like immunity proteins to prevent self-killing. This effector–immunity complex have been characterised in *Salmonella enterica* Typhimurium, *Enterobacter cloacae*, and *S. marcescens* DB10 (SmDB). The latter possesses two effector–immunity loci within the same TSS6 cluster, namely the Tae4-like Ssp1 and Ssp2, and the Tai4-like immunity proteins Rap1a, Rap1b, Rap2a, and Rap2b (English et al., 2012). The Tai4-like sequence identified as part of the *Orius* accessory genome displayed sequence similarity to SmDB Rap2a, and in close vicinity three Rap-like and one Ssp-like protein-encoding genes were identified, orthologous to SmDB Ssp and Rap proteins (**Figure 6**).

The Ssp-like and Rap-like sequences from a single representative *Orius*-derived *Serratia* genome (OLFL2) were used as query to interrogate the Ssp–Rap loci in related genomes, in order to assert the degree of sequence conservation across orthologs. Comparison of this genetic locus in the available genomes of the “SCBI-complex” and *S. marcescens* DB11 (same species as DB10) revealed striking differences. In contrast to the locus composition in SmDB, the *Orius*-derived *Serratia* sp. encodes only one effector protein Ssp2, more similar to SmDB Ssp2 (45% identity) than SmDB Ssp1 (30%), and three Rap-like immunity proteins. The latter were annotated as Rap1b, Rap2a, and Rap2b based on sequence similarity to the corresponding SmDB orthologs. This locus composition is conserved across the *Orius*-derived *Serratia* sp. and partially in *S. ureilytica*.

The locus composition and architecture differs across the rest of the “SCBI-complex” genomes (**Figure 6**), mostly in terms of number of Ssp-like and Rap-like encoding genes. Interestingly, most of the “SCBI-complex” genomes are devoid of Ssp1 and Rap1a coding sequences, except for two strains; while *Serratia* sp. SCBI and *S. marcescens* 90-166 lack a gene encoding for an Ssp effector protein. An additional interesting feature is the occurrence of two copies of *rap2a* in five genomes of the “SCBI-complex,” with one copy displaying close sequence homology (>80% identity) to its corresponding ortholog in OLFL2, while the other is only marginally similar (∼30% sequence identity). The occurrence of this trait in five genomes strongly suggests that the extra *rap2a* copy in those genomes may have originated from a gene duplication event, and probably constitutes a new sub-family within the Rap-like proteins.

Close examination of the sequence identity across the range of sequences examined demonstrated that, except for *S. ureilytica*, the Ssp2 sequence from the *Orius*-derived *Serratia* sp. strains differs substantially from orthologs encoded by the “SCBI-complex” genomes, well below the homology threshold used to define the accessory genome (95% similarity). Similarly, Rap2b is well conserved (>95% identity) in the *Orius*-derived isolates, *Serratia* sp. SCBI, and *S. marcescens* FDAARGOS 62, but its sequence conservation decreases below the threshold in the rest of the genomes composing the SCBI complex. Rap1b is only encoded by the *Orius*-derived isolates and *S. ureilytica*, but absent from the rest of the genomes in the SCBI clade (**Figure 6**). The above considerations highlight the fact that relaxing the stringency of the conditions used to generate the *Orius* accessory genome, allowing for the occurrence of sequences in all 13 *Orius*-derived isolates and one or two extra genomes from the SCBI complex, would have resulted in the inclusion of additional Ssp and Rap sequences as part of the *Orius* accessory genome.

## Discussion

The studies of microbial symbionts associated to insect hosts have been biased towards the characterisation of primary or obligate symbionts in insect species prioritised by their role as vectors for human pathogens or agricultural pests. Similarly, the nutritional benefits of the symbiotic association or the manipulation of host’s reproduction have defined paradigms in the experimental strategies used to explore symbiosis in insects; as demonstrated by the knowledge accumulated in relation to *B. aphidicola* and *Wolbachia* sp. The accessibility of next generation sequencing platforms has impacted the way in which insect–bacteria symbiosis is studied, with a plethora of reports describing symbionts at the genome level, across a wide range of insect species. However, most studies make use of single laboratory propagated populations or specimens obtained from single field collections, rather than attempting to generate population wide profiles not limited at a particular host species, but expanding onto related ones to provide characterisation of symbiotic associations at host genus level.

This study exploited a comprehensive collection of *O. laevigatus* populations originating in Spain, Italy, and Greece, together with populations of the related species *O. pallidicornis, O. niger, O. albidipennis*, and *O. majusculus*. Molecular phylogeny based on COI sequences revealed a close evolutionary relationship between *O. niger* and *O. pallidicornis*, indicating that *O. pallidicornis* can be considered a cryptic species within *O. niger*. The existence of *O. niger* cryptic species has been proposed based on COI haplotype diversity (Raupach et al., 2014), although it contrasts with a similar study using the nuclear internal transcribed spacer-1 (ITS-1) that failed to detect distinct *O. niger* lineages (Gomez-Polo et al., 2013). The inability of *O. pallidicornis* to propagate in captivity due to its strict nutritional requirement for pollen from *E. elaterium* sets this species apart from the omnivorous *O. niger*, and adds support to the interpretation of two different albeit closely related species. Further phylogenetic profiling including additional genetic markers will assert the evolutionary relationship between these species, and the time scale on which the segregation of these species took place.

Isolation of culturable bacteria from whole insect macerates, followed by whole-genome sequencing and assembly, revealed three predominant species colonising the *Orius* species under study. Two of these putative symbiotic bacteria, belonging to Erwiniaceae and Microbacteriaceae, are likely to be the first representatives of new species. Despite the sequence similarity shown by their 16S rRNA gene sequences, classifying them as members of the *Erwinia* and *Leucobacter* genera, respectively, a more thorough scrutiny using a phylogenetic reconstruction composed of a concatenation of 400 protein sequences demonstrated that these *Orius*-derived isolates grouped within strongly supported independent clades, clearly segregated from *Erwinia* sp. and *Leucobacter* sp. Comparison to taxonomically related species using digital DNA:DNA hybridisation (GGDC) failed to identify genomes similar enough to be classified as the same species, which further supports the proposal of them being new species in need of taxonomic classification. However, the lack of available genome sequences from the same species as our isolates prevented further genome comparison studies. In the absence of reference genomes belonging to these species, attempting pangenome studies would entail comparing to genomes from different, albeit related species, which would be procedurally incorrect to draw species-related information. Our observations provide the foundations for future studies exploring the symbiotic association of these bacterial species with other *Orius* species or additional insect genera.

The third predominant species isolated was classified as *Serratia* sp. based on 16S rRNA gene sequence similarity, and indeed MLSA analysis grouped these isolates within the *Serratia* sp. SCBI complex. Further refinement based on GGDC confirmed the MLSA results, that the *Orius*-associated *Serratia* sp. are sufficiently similar to genomes of the *Serratia* sp. SCBI complex to be considered the same species. However, none of the genomes within this complex is similar enough to be considered the same species as other *S. marcescens* (of which there are over 300 available genome sequences). Similarly, the SCBI complex member *S. ureilytica* has been shown to be distinct enough from *S. marcescens* by DNA–DNA hybridisation (Bhadra et al., 2005). Our brief exploration into the accuracy of the classification using GGDC indicates that the taxonomic assignment of strains as *S. marcescens*, probably historically derived from 16S rRNA gene sequences, is inaccurate in several cases.

We suggest that the broad *S. marcescens* classification is revised and curated using GGDC comparisons, to confidently segregate distinct species and establish reference genomes for comparative genomics studies. This is particularly relevant in light of widespread acceptance of *S. marcescens* strains as plant, insect, and human pathogens. For instance the human pathogens *S. marcescens* strains SM39 and SmUNAM836 are sufficiently distinct from *S. marcescens* WWW4 or DB11 to be considered different species. This taxonomic uncertainty also influences the exploitation of pangenome analyses to link *Serratia* sp. genes to lifestyle, as exemplified by the proposed association of *Serratia* sp. SCBI genes to functions related to an entomopathogenic complex association, as interpreted from comparisons between *Serratia* sp. SCBI, *S. marcescens* DB11, and *S. nematodiphila* (Abebe-Akele et al., 2015), all of them different species according to our MLSA and GGDC analyses. Surely, performing genome wide comparisons across closely related but distinct species compromises the value of the observations reported. Therefore, we propose the adoption of routine GGDC analyses as part of genome sequence deposition in databases to ensure that taxonomic classification is standardised using a readily available online tool.

A remarkable finding from this study is the conserved association of the predominant facultative symbionts described with several *Orius* species. This association was confirmed by performing genome-specific PCR across all populations under study, and further confirmed in field collected or commercially available specimens. The symbiotic association between these microorganism and *Orius* sp. is certainly demonstrated by both the wide range of geographical locations where the host populations originate and host species range tested. This approach of examining insect populations collected at diverse locations, together with the inclusion of related host species, should be routinely implemented while performing similar studies in order to provide a comprehensive view of the true extent of symbiotic associations between bacteria and insect hosts.

The inability to detect the *Leucobacter* sp. Orius strains in non-lab reared insects is puzzling but can be explained by low bacterial titres in these specimens and therefore insufficient DNA recovery for successful PCR. The artificial diet and environmental conditions provided during lab rearing may have promoted population enrichment for this otherwise minority symbiont, resulting in its isolation from insect homogenates. Although the closer identified relative to *Leucobacter* sp. Orius is the nematode-associated *Leucobacter* sp. AEAR, it is unlikely that the presence of the former in *Orius* sp. is due to systemic nematode infection in the lab-reared specimens. The COI PCR primers used within this study are generic enough to amplify the homologs target sequence from Nematoda, and the sequences derived from COI amplifications did not share similarity to available COI sequences from nematode origin. Additionally, on-going next-generation sequencing of the *Orius*-derived COI amplicons described here have not resulted in the detection of COI sequences from Nematoda (unpublished), further supporting the observation that *Leucobacter* sp. Orius are hosted by the insects tested.

Various *Leucobacter* sp. have been isolated from several insects hosts like the non-biting midges *Chironomid* sp. (Laviad et al., 2015), the scarab beetle *Holotrichia oblita* (Zhu et al., 2016), and *Anopheles gambiae* (*Leucobacter* sp. Ag1, BioSample: SAMN03481186). Based on this evidence, a facultative symbiotic association between *Leucobacter* sp. Orius and its insect host is therefore feasible. Once more genomes from this species become available, pangenome analyses will lead to the identification of genes and gene networks required for the facultative symbiotic lifestyle.

The availability of same species genome sequences allowed the extensive analysis of the *Serratia* sp. isolates from *Orius* sp. hosts. Despite the close genome sequence similarity across the isolates, the characterisation of genomic islands permitted their segregation into putative independent strains, suggesting that despite their common origin independent lineages are emerging within their respective host populations. Indeed, GI-based hierarchical clustering of these genomes closely resembles the host phylogenetic relationship. The close sequence conservation, together with the association of these *Serratia* strains to various *Orius* species, suggests a host–symbiont association established in the last common ancestor to present day European *Orius* species, albeit not reflected by genome size reduction. An alternative explanation is to interpret this facultative symbiosis as a consequence of the routine acquisition of this *Serratia* species from environmental sources. The failure to isolate other *Serratia* species from the *Orius* sp. specimens tested adds weight to the interpretation that this *Orius*–*Serratia* association is an ancient symbiotic event. However, expanding this study to *Orius* species from distant geographical locations and archived specimens is needed to determine the extent of the symbiotic relationship.

*Serratia* species possesses the ability to colonise a variety of niches and are both successful mutualistic symbionts and opportunistic pathogens. In particular their ability to persist in symbiosis with insect-associated nematodes has been demonstrated (Petersen and Tisa, 2013). The pangenome analyses described here clearly associate genes from the accessory genome of *Serratia* sp. colonising *Orius* species with the insect symbiosis trait, and segregates them from similar species from the SCBI complex, including a known nematodes symbiont like *Serratia* sp. SCBI. Annotation of these accessory genes failed to conclusively assign functional roles related to the symbiotic lifestyle. However, the occurrence within this *Orius* symbiont accessory genome of TSS6 Tae4–Tai4 effector–immunity cognate partners suggests that the establishment of a symbiotic lifestyle requires molecular mechanisms ensuring successful interspecies competition. Indeed, products secreted by TSS6 systems encoded by *Serratia* sp. have been shown to confer antimicrobial properties targeting microbial competitors to ensure survival in polymicrobial environments (Murdoch et al., 2011; English et al., 2012; Srikannathasan et al., 2013). The host symbiont association between *Orius* sp. and the *Serratia* isolates described may have driven the acquisition and specialisation of strain-specific TSS6 effector–immunity partnerships to preserve niche colonisation from closely related, invading environmental or pathogenic *Serratia* species. In particular the sequence divergence of “orphan” Tai4 immunity proteins (Rap2a, Rap2b) in *Orius*-associated *Serratia*, without the presence of cognate Ssp-like effector, suggests that a trait has evolved to expand the resistance range against effector proteins secreted by invading bacteria, as suggested for other bacterial species (Srikannathasan et al., 2013).

The results described earlier demonstrate the existence of a facultative symbiotic association between European *Orius* species collected across a wide range of geographical locations, and a new *Serratia* species closely related to *Serratia* sp. SCBI and other species within this complex. We propose to refer to this group of isolates *Serratia* sp. Orius, until a thorough taxonomic classification is provided. These findings agree with a widespread symbiotic or pathogenic lifestyle embraced by various species comprising the “SCBI complex,” namely insect and nematode pathogens (*Serratia* sp. SCBI and TEL), plant associated (*S. marcescens* 90-166, *Serratia* sp. RSC-14), and human pathogens (*S. marcescens* BIDMC81, BIDMC50, and FDAARGOS62). The latter in particular is extremely relevant in lieu of the use of *Orius* species as pest control agents in crops destined for human consumption, and at least one reported case of targeted biting of humans by *Orius majusculus* (Kampen and Werner, 2011). The genome sequence comparisons described earlier suggest that the *Serratia* sp. Orius isolates are a distinct sub-species from those shown to be pathogenic to humans, and therefore expressing concerns over the safety of their insect hosts in IPM applications is premature. Nevertheless, regulatory agencies monitoring the use of biological control should engage in discussion with the wider academic community to explore the feasibility of implementing microbiome characterisation as part of the standard quality control measures governing the use of insects as pest control agents.

## Author Contributions

XC performed molecular microbiology tasks and genome sequence analyses. MH performed genome sequencing, genome assembly, pangenome analyses, contributed to experimental design, and manuscript preparation. PB, JM, and VB collected and propagated insect populations, performed taxonomic classification, and contributed to molecular biology tasks and manuscript preparation. PF performed molecular phylogeny analyses, contributed to experimental design, and manuscript preparation. PD contributed to manuscript preparation. RD supervised the project, designed experiments, performed genome sequence and molecular phylogeny analyses, and drafted the manuscript. All authors read and approved the final manuscript.

## Conflict of Interest Statement

The authors declare that the research was conducted in the absence of any commercial or financial relationships that could be construed as a potential conflict of interest.

## Footnotes

1 http://ggdc.dsmz.de/distcalc2.php

2 http://weizhongli-lab.org/cdhit_suite/cgi-bin/index.cgi?cmd=cd-hit-est
