# Supplementary figures and images for: Comparative Genomics of Facultative Bacterial Symbionts Isolated from European Orius Species Reveals an Ancestral Symbiotic Association
Source: Front Microbiol. 2017 Oct 10;8:1969. doi: 10.3389/fmicb.2017.01969 (PMC5641365; doi:10.3389/fmicb.2017.01969)

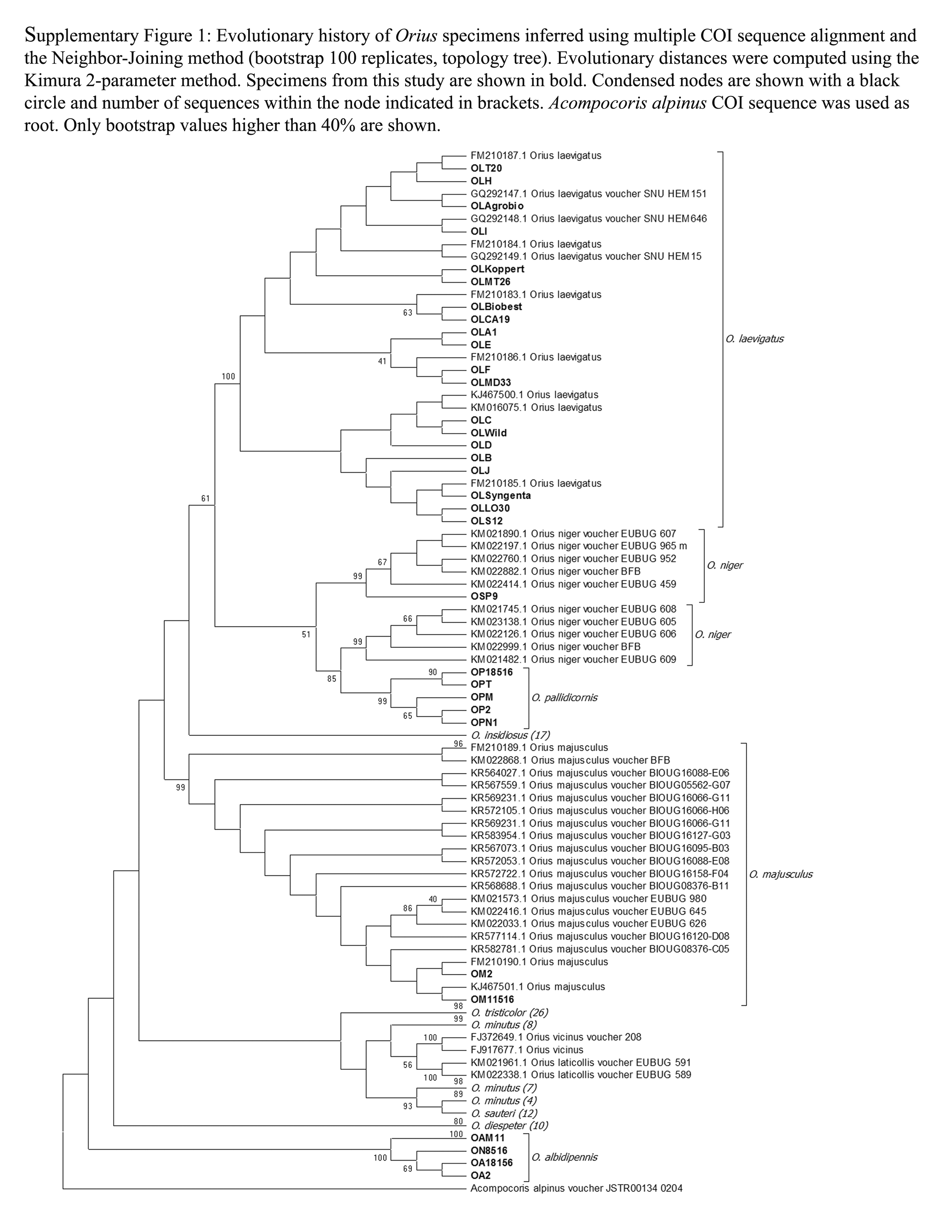

Supplement: Supplementary file 9 [file Image_1.TIF]

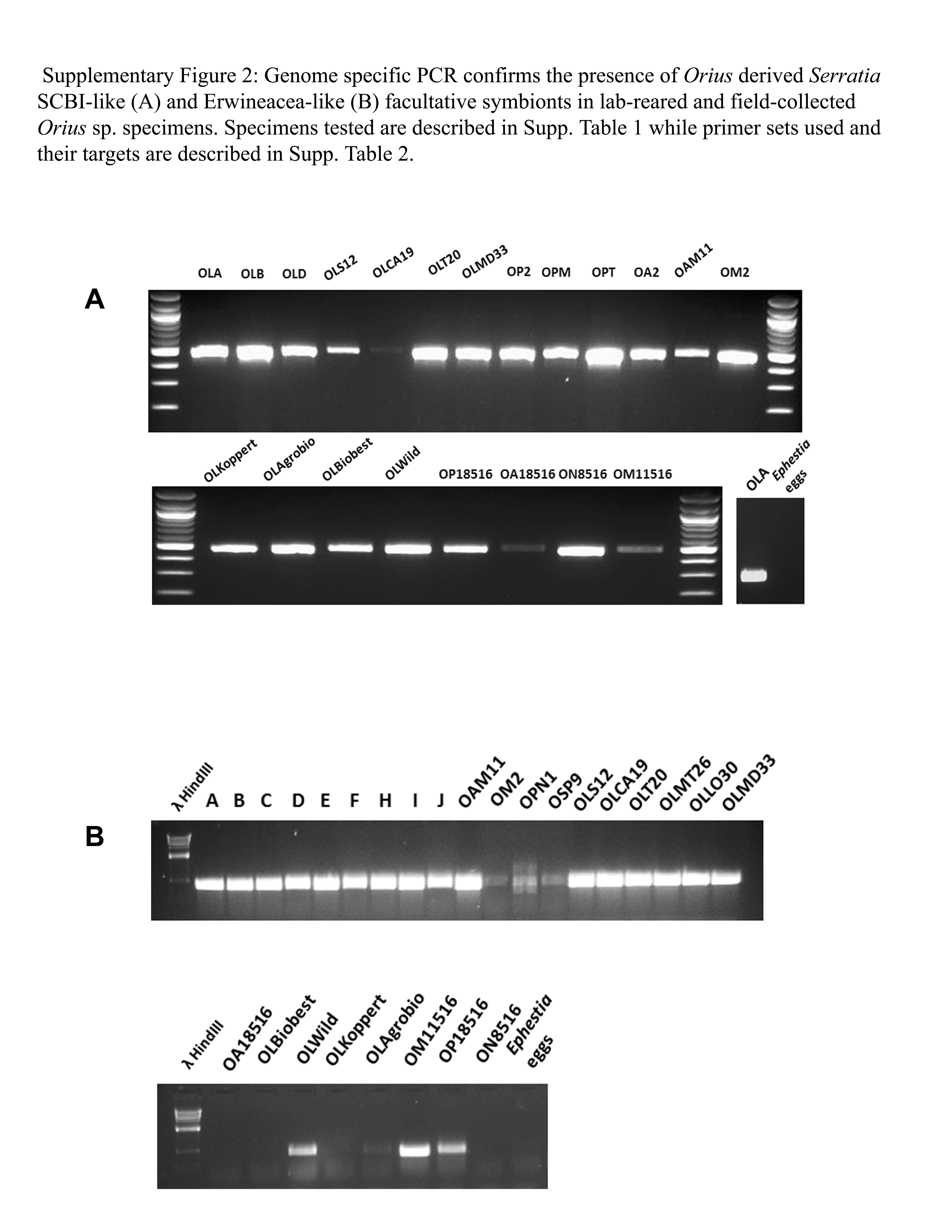

Supplement: Supplementary file 10 [file Image_2.TIF]

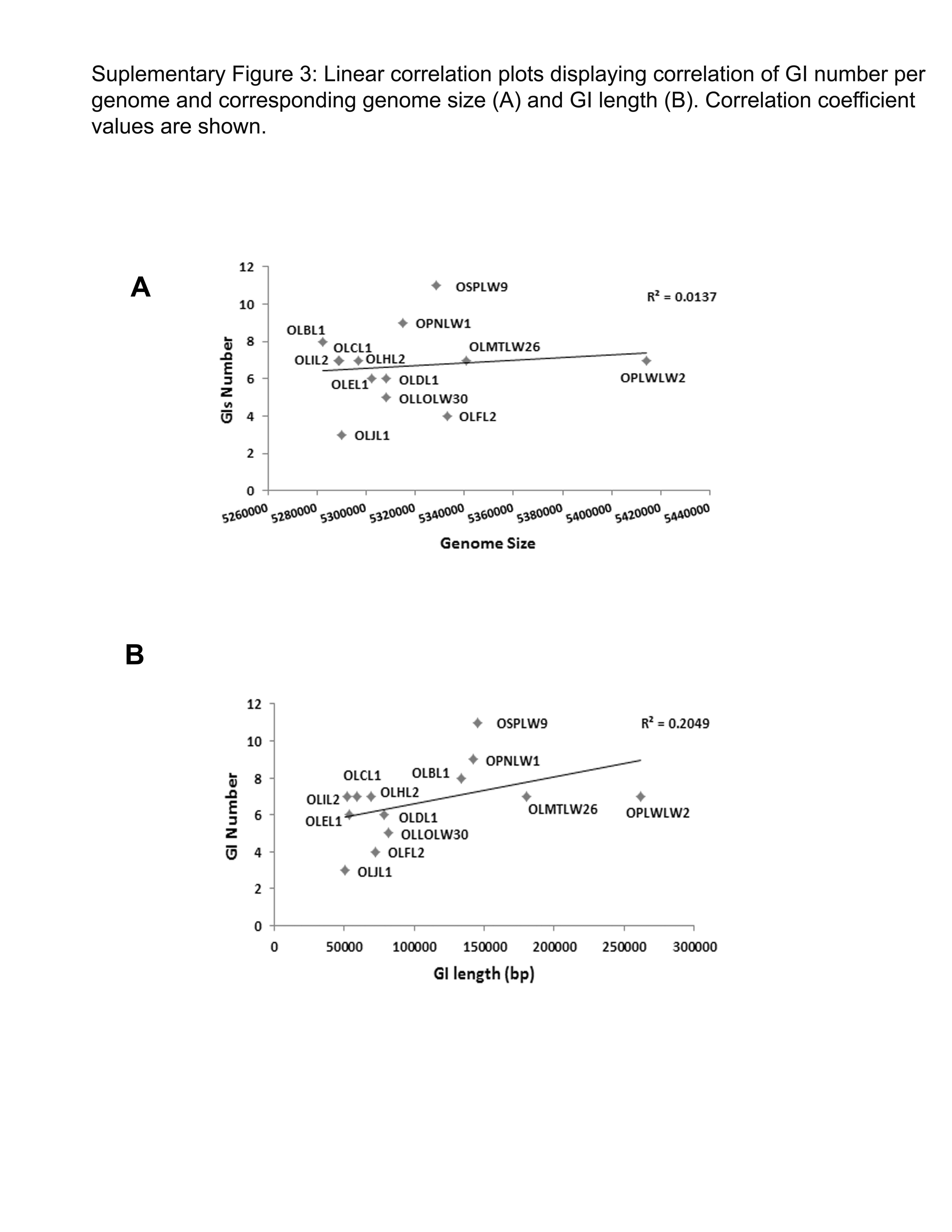

Supplement: Supplementary file 11 [file Image_3.TIF]
